# Closure of macular hole secondary to ischemic hemi-central retinal vein occlusion by retinal photocoagulation and topical anti-inflammatory treatment

**DOI:** 10.1007/s10103-020-03133-9

**Published:** 2020-08-21

**Authors:** Teru Asato, Norihiro Nagai, Misa Suzuki, Atsuro Uchida, Toshihide Kurihara, Norimitsu Ban, Sakiko Minami, Hajime Shinoda, Kazuo Tsubota, Yoko Ozawa

**Affiliations:** 1 Department of Ophthalmology, Keio University School of Medicine, 35 Shinanomachi, Shinjuku-ku, Tokyo, 160-8582 Japan; 2 Laboratory of Retinal Cell Biology, Keio University School of Medicine, 35 Shinanomachi, Shinjuku-ku, Tokyo, 160-8582 Japan; 3 Department of Ophthalmology, St. Luke’s International Hospital, 9-1 Akashi-cho, Chuo-ku, Tokyo, 104-8560 Japan; 4 St. Luke’s International University, 9-1 Akashi-cho, Chuo-ku, Tokyo, 104-8560 Japan

To the Editor:

We report a case of full-thickness macular hole (MH) secondary to hemi-central retinal vein occlusion (CRVO) that was closed after retinal photocoagulation to the non-perfusion area (NPA) and anti-inflammatory treatment by topical bromfenac sodium hydrate.

**Fig. 1:**
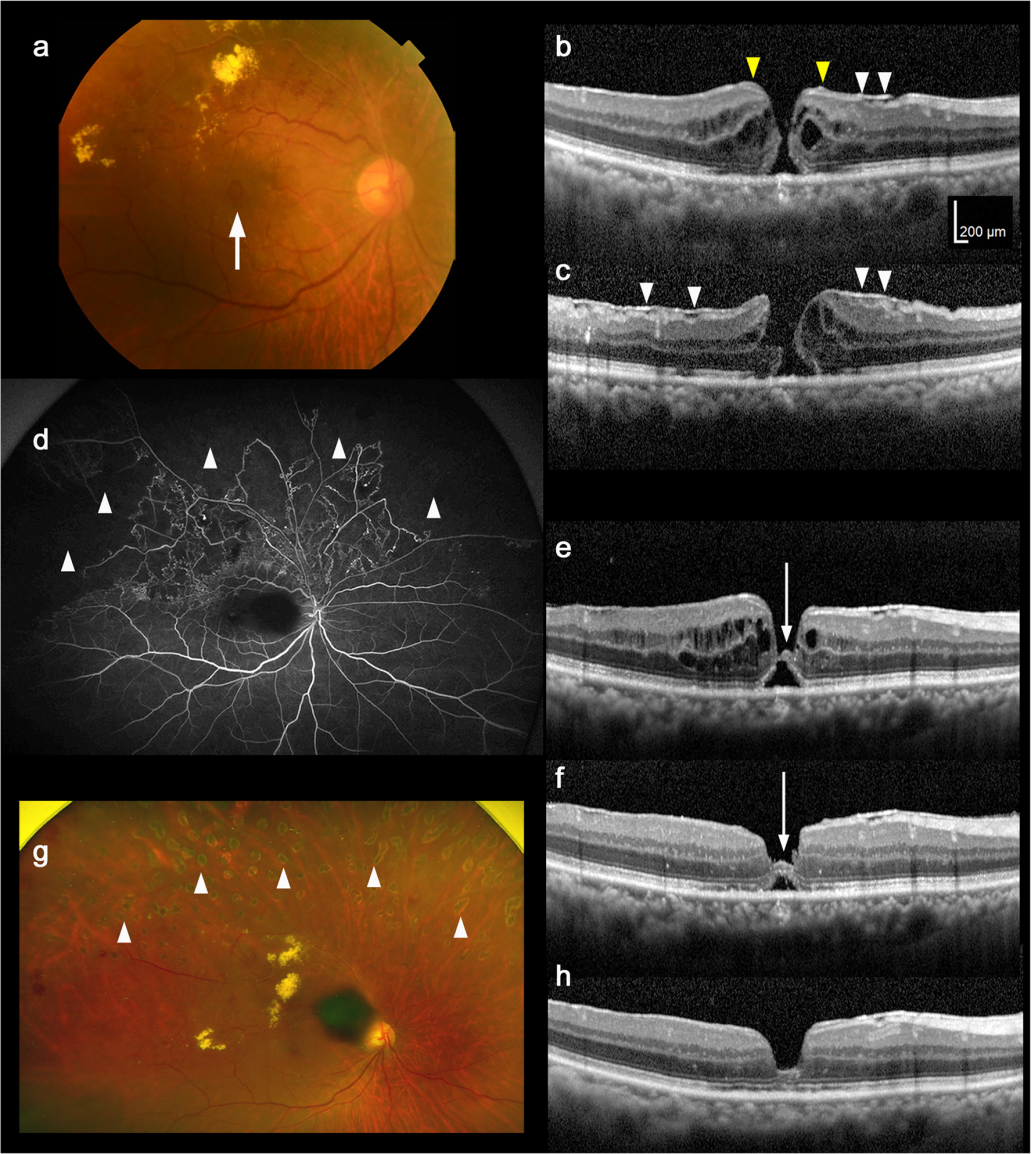
Clinical course of the eye with the macular hole (MH) secondary to hemi-central retinal vein occlusion (CRVO) that achieved closure by retinal photocoagulation and topical bromfenac sodium hydrate. Fundus photograph (**a**, arrow) and optical coherence tomography (OCT) image (**b**, **c**) at first visit (August 2019) showing a full-thickness MH. Note that there was formation of epiretinal membrane (ERM) (white arrowheads) and lamellar hole-associated epiretinal proliferation (LHEP)-like material (yellow arrowheads). **d** Fluorescein angiography at first visit showed vast area of non-perfusion (white arrowheads) and vascular inflammation. OCT images one (**e**) and two (**f**) months after photocoagulation showed formation of bridging element (arrow) in the MH. The thickened element included recovered external limiting membrane (ELM) (**f**). Fundus photograph (**g**) and OCT image (**h**) 3 months after photocoagulation. Laser scars were evident in the non-perfusion area (**g**, arrowheads). MH successfully closed (**h**). Note that the dark spot in **g** is not a hemorrhage but an artifact in the fundus photograph at the time of recording (California, Optos, Inc., MA).

In July 2019, a 72-year-old Japanese man was referred to the Medical Retina Division of Keio University Hospital (Tokyo, Japan) due to blurred vision in his right eye for 6 months. He had a history of unilateral nephrectomy. He had hemi-CRVO (Fig. 1a) and a full-thickness MH (Fig. 1a–c) with posterior vitreous detachment in his right eye. The optical coherence tomography (OCT) image showed epiretinal membrane (ERM) (Fig. 1b, c white arrowheads) and lamellar hole-associated epiretinal proliferation (LHEP)-like material (Fig. 1b yellow arrowheads) around the MH edge. His best-corrected visual acuity (BCVA) was 0.15 (0.823 LogMAR). Fluorescein angiography (FA) showed extensive NPA and vascular leakage in the affected area (Fig. 1d); he was diagnosed with ischemic hemi-CRVO and underwent retinal photocoagulation to prevent neovascularization. Topical bromfenac sodium hydrate was prescribed and continued throughout the course to reduce retinal vein occlusion (RVO)-related inflammation and photocoagulation-related transient inflammation [1]. In August 2019, a tiny bridging element appeared between the MH edges in the OCT image (Fig. 1e arrow); his BCVA improved slightly to 0.3 (0.523 LogMAR). In September 2019, the bridging element thickened and an external limiting membrane (ELM) was formed (Fig. 1f arrow); his BCVA improved to 0.7 (0.155 LogMAR). In November 2019, the ellipsoid zone recovered, and the MH closed (Fig. 1g, h); his BCVA recovered to 0.8 (0.100 LogMAR). Laser scars were evident in the NPA (Fig. 1g arrowheads).

MH is generally formed by tractional forces at the vitreoretinal interface and treated by surgical removal of these forces; pars plana vitrectomy is typically used. Although the affected eye had secondary ERM, which may have caused tractional forces, and LHEP-like material, which was composed of migrated Müller glial cells that could be induced by ERM-associated traction [2], the MH was closed without surgical removal of these materials. Because retinal photocoagulation reduces hypoxia-induced excessive vascular endothelial growth factor (VEGF) production in the retinal NPA [3], photocoagulation most likely reduced the retinal inflammation. Together with the topical non-steroidal anti-inflammatory drug (NSAID), this may have helped in closing the MH. A previous report showed a full-thickness MH formation after anti-VEGF therapy to treat macular edema due to RVO [4]. However, to the best of our knowledge, this is the first report of a full-thickness MH secondary to ischemic hemi-CRVO that was closed after photocoagulation and topical NSAID application, without using pars plana vitrectomy.

An earlier report described an idiopathic MH with multiple recurrences and spontaneous closures that involved retinal periphlebitis, as recorded by FA during MH recurrence, and closure was achieved by topical steroid [5]. Thus, inflammation could be involved in certain types of MH; the unique point of the affected eye in the current case was that closure was most likely achieved by photocoagulation to the NPA. Moreover, Hayashi et al. also reported that a bridging element was found before MH closure [5]. The development and thickening of the bridging element preceded the recovery of ELM (Fig. 1e) and ellipsoid zone, and MH closure (Fig. 1h) in the current case, suggesting that this finding may be a predictor of MH closure.

The MH in ischemic hemi-CRVO was most likely the result of associated retinal inflammation since closure was achieved by anti-inflammatory treatment with retinal photocoagulation and topical NSAID. A bridging element between the MH edges recorded by OCT could be a sign of closing MH and may help in determining the treatment plan.
